# Effect of continuation of antiplatelet therapy on survival in patients receiving physician home visits

**DOI:** 10.1186/s12877-019-1394-6

**Published:** 2019-12-23

**Authors:** Yasuhiro Osugi, Teruo Ino, Daiki Kobayashi, Mitsunaga Iwata, Kanichi Asai

**Affiliations:** 1 Department of Community Based Medicine, Fujita Health University, Toyoake, Japan; 2 Toyota Regional Medical Center, Toyota, Japan; 3 Division of General Internal Medicine, Department of Medicine, St. Luke’s International Hospital, Tokyo, Japan; 4 Center for Clinical Epidemiology, St. Luke’s International Hospital, Tokyo, Japan

**Keywords:** Aspirin, Home visiting care, Mortality, Antiplatelet therapy, Survival patients

## Abstract

**Background:**

Little is known about the effects of continued antiplatelet therapy in patients who receive physician home visits. This study aimed to evaluate the association of survival with the continuation of antiplatelet drugs in patients who received physician home visits.

**Methods:**

A retrospective cohort study was conducted in a teaching hospital in Toyota, Japan, from April 2015 to October 2018. All patients who received home visits by physicians from the department of Family Medicine of the hospital were included. The primary outcome was the difference in all-cause mortality between patients who were taking antiplatelet drugs and those who were not. The Cox proportional hazards model was applied, adjusted for the patient’s demographic features, activities of daily living, comorbidities, and primary disease requiring home care.

**Results:**

A total of 815 patients were included, of whom 61 received antiplatelet drugs (*n* = 42 for aspirin, *n* = 17 for clopidogrel, and *n* = 8 for cilostazol) and 772 received no antiplatelet drugs. The mean age of the patients was 78.3 years, 409 (49.1%) were male, and 314 (37.7%) had end-stage cancer. During a median follow-up period of 120 days (interquartile range, 29–364), 54.3% of the patients died. Compared with patients not taking antiplatelet drugs, patients taking antiplatelet drugs had a better outcome (*p* <  0.01, log-rank test) and a significantly lower hazard ratio (0.34; 95% confidence interval, 0.17–0.65; Cox proportional hazards regression).

**Conclusions:**

The continuous prescription of antiplatelet drugs may have beneficial effects on mortality among patients who receive physician home visits.

## Background

Aspirin is used widely in preventing several diseases. As secondary prevention, aspirin has a significant impact on cardiovascular mortality, acute coronary diseases, and stroke [1, 2]. Studies have reported that aspirin reduces the risk of cardiovascular disease and mortality from cardiovascular disease by 20% in patients with established cardiovascular diseases. A meta-analysis reported that aspirin as secondary prevention reduced the risk of serious vascular events by 18% and the risk of fatal stroke by one-fifth [3]. In contrast to secondary prevention, the evidence for primary prevention of several diseases by aspirin is mixed. Previous meta-analyses suggested that aspirin potentially reduced all-cause mortality by 6–8%, but the effect was not statistically significant [4, 5]. With regard to primary prevention of myocardial infarction, randomized, controlled trials failed to show a benefit of aspirin [6, 7], whereas a meta-analysis showed significant benefits [5]. Therefore, the indication for aspirin should be evaluated before prescribing it.

The evidence for the benefits of aspirin for the prevention of all-cause mortality, cardiovascular disease, and stroke becomes more complicated when patient characteristics are considered. In vulnerable patients, such as elderly people or those with cancer, the number or severity of side effects of aspirin may exceed the benefits. An observational study reported that elderly patients receiving aspirin-based antiplatelet therapy without a proton pump inhibitor had substantially higher risks of disabling or fatal gastrointestinal bleeding and intracranial hemorrhage [8]. It has been suggested that most patients with end-stage cancer should stop using aspirin to prevent cardiovascular disease, although evidence for this recommendation is lacking [9]. Thus, the patient’s condition, in addition to the diseases targeted for prevention, should be considered when prescribing aspirin or other antiplatelet drugs.

The new antiplatelet drugs clopidogrel and cilostazol are used widely for the secondary prevention of cerebrovascular diseases [10] in place of, or in combination with, aspirin. The effects of these drugs on survival have not yet been reported. We therefore conducted a study to test whether these drugs have beneficial effects on survival in patients receiving home visits by physicians.

Patients who receive physician home visits are substantially more vulnerable than the general population. The Japanese Ministry of Health, Labour, and Welfare reported that more than half the patients receiving physician home visits had dementia and more than 10% had cancer [10]. The benefits of prescriptions for patients with dementia are questionable [11], because they may misuse drugs, resulting in unpredictable side effects. Patients with dementia may be more disabled than patients who visit hospitals, because of physical, mental, or social problems that make it difficult to visit hospitals. Patients with disability using antiplatelet therapy may be subject to traumatic bleeding from falls. Thus, various comorbidities may affect the prescription of aspirin in patients receiving home healthcare. A detailed evaluation of patients receiving physician home visits is needed to determine the indications of antiplatelet drugs.

The aim of this study was to evaluate the association of survival with the continuation of antiplatelet drugs in patients who received physician home visits.

## Methods

### Patients

We conducted a retrospective cohort study in Toyota Regional Medical Center, Toyota, Japan, from April 2015 to October 2018. We included all patients who received home visits from physicians of the hospital’s Department of Family Medicine. The patients were visited by a physician at least twice a month and by a nurse an average of six times a month. They were also visited separately by a pharmacist twice a month, in collaboration with physicians and nurses to deliver drugs and give instructions to improve drug adherence. Most of the patients were discharged from nearby acute-care hospitals to home, and others were referred from local general practitioners. We excluded patients who had agreed to receive physician home visits, but never received due to admission to hospital or care facilities, or death before first visit. We compared the mortality of patients who were taking antiplatelet drugs with that of patients who were not taking antiplatelet drugs.

### Ethical approval

The ethical committee of the hospital approved this study (approval number: 30-kenrin10).

### Antiplatelet drugs

We defined patients who were taking antiplatelet drugs as those who were taking any dosage of aspirin, clopidogrel, or cilostazol at the beginning of the physician home visits (*n* = 61). In most cases, antiplatelet treatment was continued throughout the observation period or until the end of life care, unless it was stopped because of gastrointestinal symptoms. The primary reasons for the prescription of antiplatelet drugs were obtained from electronic medical records at the hospital or referral documents from other hospitals. Among the 61 patients who were taking antiplatelet drugs, 42 were taking aspirin, 17 clopidogrel, and 8 cilostazol, while 5 were taking both aspirin and clopidogrel and 1 was taking both aspirin and cilostazol. Prior to the physician home visits, these antiplatelet drugs had been prescribed by a hospital doctor or a general practitioner. All information was extracted from electronic medical records in the hospital and no missing data was identified.

### Outcomes

Our primary outcome was all-cause mortality during the study period. Patients who died at home or who were admitted to and subsequently died in the hospital were considered treatment failures. Those who terminated physician home visits or were admitted to other hospitals were considered as lost to follow-up.

### Covariates

We obtained patient information, including demographic features, activities of daily living (ADL), primary disease requiring physician home visits, and comorbidities. The patient’s ADL was evaluated according to the following abilities: ambulation (confined to bed, confined to wheelchair, walks with assistance, walks independently), eating (unable to eat, eats with assistance, eats independently), and toileting (needs complete assistance, needs some assistance, independent). The primary diseases requiring physician home visits were categorized based on beta clinical classification software for ICD-10-CM/PCS according to the Healthcare Cost and Utilization Project [12]. Comorbidities were evaluated according to the Charlson comorbidity index [13].

### Statistical analysis

First, we compared baseline characteristics, ADL, primary disease requiring physician home visits, and comorbidities between patients who took antiplatelet drugs and those who did not by the *t-*test and the chi-squared test. Then, we compared all-cause mortality between the two patient groups by Kaplan–Meier curves and the log-rank test. We next analyzed outcomes by a multivariate Cox proportional hazards model after adjusting for covariates, which could affect on our outcomes, including survival rates. Before performing Cox proportional hazard model, we examined proportional hazard assumption based on log-log plot and Kaplan-Meier curves with predicted survival plots. We applied different models with different covariates to confirm the results. Model 1 included age, gender, and ADL for adjustment; model 2 included primary disease requiring physician home visits in addition to model 1; and model 3 included the Charlson comorbidity index (including cancer bearing status) in addition to model 2. We evaluated the outcome between those who took any antiplatelet agents and those who didn’t, and between those who took each antiplatelet agent and those who didn’t. Patients without each antiplatelet drug included those who took other antiplatelet drugs than the main drug. (e.g. patients without aspirin composed from those who didn’t take any antiplatelet drugs and those who took either clopidogrel or cilostazol.) As sub-analyses, we examined all-cause mortality between the two patient groups separately among end-stage cancer patients and among patients without end-stage cancer. In addition, we performed sensitivity analyses by excluding patient who lost follow up or died within 2 weeks to mitigate potential reverse causality that physicians might not prescribe antiplatelet drugs because of patients’ serious/ life-threatening condition. All analyses were performed by Stata 14.0 (Stata Corp., College Station, TX, USA).

## Results

After excluding 18 patients who agreed to physician home visits, but not actually received due to admission to hospitals or death, a total of 815 patients were included, of whom 61 (7.5%) received antiplatelet drugs (*n* = 42 for aspirin, *n* = 17 for clopidogrel, and *n* = 8 for cilostazol) and 754 received no antiplatelet drugs. The mean age of the patients was 78.2 ± 12.4 [standard deviation (SD)] years, 399 (49.0%) were male, and 328 (40.3%) had end-stage cancer. Among end-stage cancer patients, 11 (3.5%) took antiplatelet drugs, whereas remaining 301 (96.5%) didn’t. Table 1 compares patient characteristics, including demographics, ADL, and primary disease requiring home visiting care between those who received antiplatelet drugs and those who did not. Those who received antiplatelet drugs were more likely to have diseases of the circulatory system (*p* <  0.01), but less likely to have neoplasms (*p* <  0.01) as the primary disease requiring physician home visits. Table 2 compares each component of the Charlson comorbidity index between those who received antiplatelet drugs and those who did not. Those who received antiplatelet drugs were more likely to have cardiovascular risk factors or diseases and less likely to have cancer. The total Charlson comorbidity index score was 2.0 ± 2.0 (mean ± SD) in patients who received antiplatelet drugs and 2.0 ± 2.2 in patients who did not; the difference was not statistically significant.

**Table 1:** Comparison of baseline demographic characteristics of patients taking and not taking antiplatelet agents

Characteristic	Taking antiplatelets (*n* = 61)	Not taking antiplatelets (*n* = 754)	*p* value	Total (*n* = 815)
Age, years (SD)	79.4 (11.6)	78.2 (12.5)	0.45	78.2 (12.4)
Male, *n* (%)	31 (50.8)	368 (48.8)	0.76	399 (49.0)
Died, *n* (%)	**16 (26.2)**	**422 (56.0)**	**< 0.01**	**438 (53.8)**
Activities of daily living				
Ambulation			0.45	
Confined to bed	2 (3.8)	42 (6.1)		44 (5.9)
Confined to wheelchair	17 (32.1)	187 (27.1)		204 (27.4)
Walks with assistance	16 (30.2)	165 (23.9)		181 (24.3)
Walkings independently	18 (34.0)	297 (43.0)		315 (42.3)
Eating			0.41	
Unable to eat	3 (5.7)	29 (4.2)		32 (4.3)
Eats with assistance	41 (77.4)	490 (70.9)		531 (71.4)
Eats independently	9 (17.0)	172 (24.9)		181 (24.3)
Toileting			0.52	
Needs complete assistance	6 (11.3)	58 (8.4)		64 (8.6)
Needs some assistance	21 (39.6)	241 (34.9)		262 (35.2)
Independent	26 (49.1)	392 (56.7)		418 (56.2)
Primary disease requiring physician home visits, n (%)				
Congenital anomalies	0 (0.0)	3 (0.4)	0.62	3 (0.4)
Diseases of the circulatory system	**25 (41.0)**	**115 (15.3)**	**< 0.01**	**140 (17.2)**
Diseases of the digestive system	23 (3.0)	0 (0.0)	0.17	23 (2.8)
Diseases of the genitourinary system	3 (4.9)	17 (2.3)	0.20	20 (2.5)
Diseases of the musculoskeletal system and connective tissue	2 (3.3)	32 (4.2)	0.72	34 (4.2)
Diseases of the nervous system and sense organs	4 (6.6)	61 (8.1)	0.67	65 (8.0)
Diseases of the respiratory system	6 (9.8)	56 (7.4)	0.50	62 (7.6)
Diseases of the skin and subcutaneous tissue	1 (1.6)	3 (0.4)	0.18	4 (0.5)
Endocrine, nutritional, and metabolic diseases	4 (6.6)	22 (2.9)	0.12	26 (3.2)
Infectious and parasitic diseases	0 (0.0)	11 (1.5)	0.34	11 (1.4)
Injuries and poisoning	1 (1.6)	27 (3.6)	0.42	28 (3.4)
Mental illness	4 (6.6)	65 (8.6)	0.58	69 (8.5)
Neoplasms	**11 (18.0)**	**301 (39.9)**	**< 0.01**	**312 (38.3)**
Unknown	0 (0.0)	18 (2.4)	0.22	18 (2.2)

Boldface indicates p value < 0.05

**Table 2:** Comparison of Charlson comorbidity index scores of patients taking and not taking antiplatelet agents

Comorbidity	Taking antiplatelets (*n* = 61)	Not taking antiplatelets (*n* = 754)	*p* value	Total (*n* = 815)
Acute myocardial infarction	**4 (6.6)**	**7 (0.9)**	**< 0.01**	**11 (1.4)**
Congestive heart failure	**14 (23.0)**	**73 (9.7)**	**< 0.01**	**87 (10.7)**
Peripheral vascular disease	0 (0.0)	2 (0.3)	0.69	2 (0.3)
Cerebral infarction	**14 (23.0)**	**33 (4.4)**	**< 0.01**	**47 (5.8)**
Cerebral bleeding	2 (3.3)	13 (1.7)	0.39	15 (1.8)
Dementia	5 (8.2)	56 (7.4)	0.83	61 (7.5)
Pulmonary disease	**9 (14.8)**	**55 (7.3)**	**0.04**	**64 (7.9)**
Connective tissue disorder	0 (0.0)	0 (0.0)	1.00	0 (0.0)
Peptic ulcer	0 (0.0)	5 (0.7)	0.52	5 (0.6)
Mild to moderate liver disease	0 (0.0)	30 (4.0)	0.11	30 (3.7)
Diabetes	**12 (19.7)**	**41 (5.4)**	**< 0.01**	**53 (6.5)**
Paraplegia	0 (0.0)	1 (0.1)	0.78	1 (0.1)
Renal disease	5 (8.2)	35 (4.6)	0.22	40 (4.9)
Diabetes with complications	0 (0.0)	2 (0.3)	0.69	2 (0.3)
Cancer	**13 (21.3)**	**321 (42.6)**	**0.01**	**334 (41.0)**
Leukemia	0 (0.0)	2 (0.3)	0.69	2 (0.2)
Malignant lymphoma	0 (0.0)	10 (1.3)	0.37	10 (1.2)
Severe liver disease	0 (0.0)	18 (2.4)	0.22	18 (2.2)
Metastatic cancer	4 (6.6)	74 (9.8)	0.41	78 (9.6)
HIV	0 (0.0)	0 (0.0)	1.00	0 (0.0)

Boldface indicates p value < 0.05

**Fig. 1:**
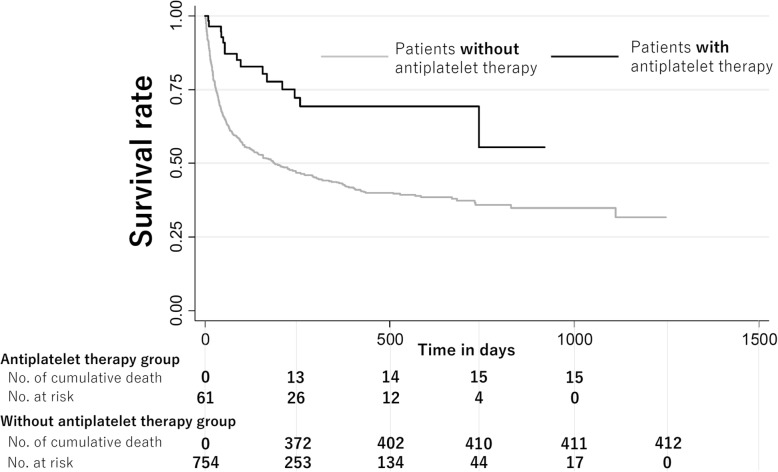
Comparison of Kaplan-Meire curves for survival rate between patients with antiplatelet drugs and those without antiplatelet drugs

**Fig. 2:**
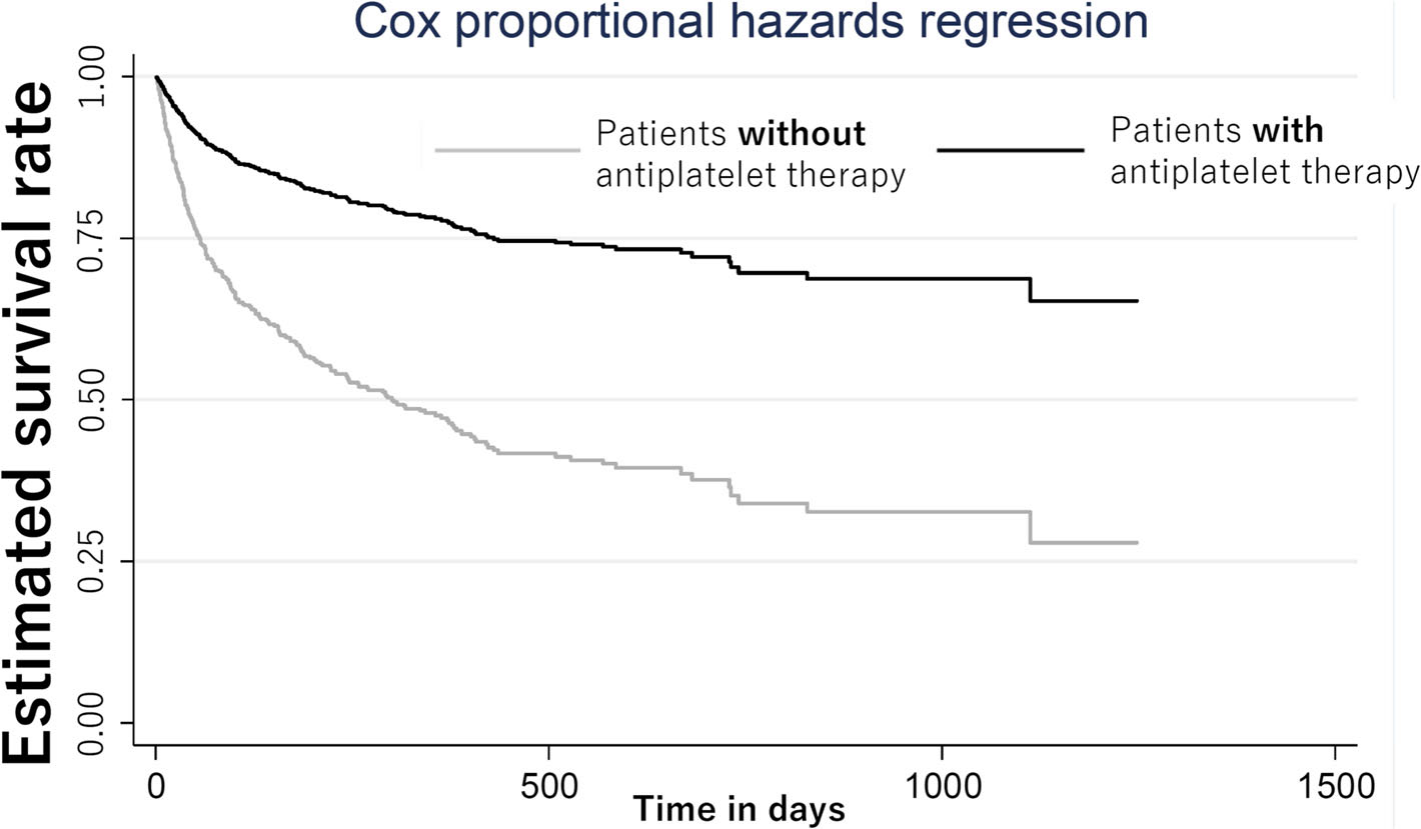
Adjusted survival curves by antiplatelet therapy status from Cox proportional hazard regression

During a median follow-up period of 114 days (interquartile range, 29–361), 438 patients (53.8%) died. Among them, 212 (48.4%) patients died due to cancer (2 (3.3%) among those who took any antiplatelet agents; 210 (27.9%) among those who didn’t take any antiplatelet agents). Figure 1 shows the Kaplan–Meier curves for patients taking and not taking antiplatelet drugs. Patients taking antiplatelet drugs had a better outcome (*p* <  0.01). Table 3 shows the hazard ratios (HRs) for mortality for patients taking and not taking antiplatelet drugs for models with different covariates (HRs for mortality of adjustment variables were shown in Additional file 1). The HR was significantly lower for those who were taking antiplatelet drugs [HR, 0.33; 95% confidence interval (CI), 0.17–0.65]. In model 3, the HR was significantly lower for those who were taking aspirin alone (HR, 0.32; 95% CI, 0.15–0.69), but not for those who were taking clopidogrel (HR, 0.48; 95% CI, 0.15–1.59) or cilostazol (HR, 0.89; 95% CI, 0.19–4.02), compared with patients who were not taking antiplatelet drugs. Figure 2 shows the adjusted survival curves for patients with and without antiplatelet therapy based on model 3. We confirmed that proportional hazard assumption was not violated, because two curves in log-log plot showed roughly paralleled and predicted survival plots were on the observed curves.

**Table 3:** Hazard ratio (HR) for mortality among patients taking antiplatelet agents compared with those not taking antiplatelet agents

Therapy	Adjusted HR (95% CI)		
	Model 1	Model 2	Model 3
Any antiplatelet therapy v.s. Without antiplatelet therapy	**0.28 (0.15–0.53)**	**0.35 (0.18–0.65)**	**0.33 (0.17–0.65)**
Aspirin v.s. Without aspirin	**0.28 (0.13–0.60)**	**0.32 (0.15–0.67)**	**0.32 (0.15–0.69)**
Clopidogrel v.s. Without clopidogrel	**0.27 (0.09–0.84)**	0.52 (0.16–1.66)	0.48 (0.15–1.59)
Cilostazol v.s. Without cilostazol	0.58 (0.14–2.33)	0.67 (0.16–2.74)	0.89 (0.19–4.02)

Model 1 includes patient’s demographic characteristics and activities of daily living (ADL) for adjustment; model 2 includes primary disease requiring home visiting care in addition to model 1; and model 3 includes the Charlson comorbidity index in addition to model 2

Patients without each antiplatelet drug included those who took other antiplatelet drugs than the main drug. (e.g. patients without aspirin composed from those who didn’t take any antiplatelet drugs and those who took either clopidogrel or cilostazol)

Boldface indicates p value < 0.05

In sub-analyses by end-stage cancer status, those who took any antiplatelet agents had significantly lower HR of mortality compared to those who didn’t take antiplatelet agents among end-stage cancer patients (HR, 0.13; 95% CI, 0.03–0.64) (Table 4). Similar to any antiplatelet agents, those who took aspirin also had significantly lower mortality (HR, 0.15; 95% CI, 0.03–0.74). In contrast, none of antiplatelet agents had significantly different HR of mortality from without antiplatelet agents among non-end-stage cancer patients.

**Table 4:** Hazard ratio (HR) for mortality among patients taking antiplatelet agents compared with those not taking antiplatelet agents, stratified by bearing end-stage cancer

Therapy	Adjusted HR (95% CI)	
	Patients with end-stage cancer	Patients without end-stage cancer
Any antiplatelet therapy v.s. Without antiplatelet therapy	**0.13 (0.03–0.64)**	0.58 (0.26–1.26)
Aspirin v.s. Without aspirin	**0.15 (0.03–0.74)**	0.52 (0.20–1.34)
Clopidogrel v.s. Without clopidogrel	—^a^	0.52 (0.15–1.83)
Cilostazol v.s. Without cilostazol	—^a^	3.41 (0.74–15.7)

Model includes patient’s demographic characteristics, activities of daily living (ADL), primary disease requiring home visiting care and the Charlson comorbidity index

Patients without each antiplatelet drug included those who took other antiplatelet drugs than the main drug. (e.g. patients without aspirin composed from those who didn’t take any antiplatelet drugs and those who took either clopidogrel or cilostazol)

^a^ Analyses could not be performed due to limited number of samples

Boldface indicates *p* value < 0.05

In our sensitivity analyses by excluding patient who lost follow up or died within 2 weeks, HR of mortality among patients receiving any antiplatelet agents compared to among patients didn’t, and that among patients receiving aspirin compared to among patients didn’t were still significantly lower (Additional file 2).

Among patients receiving antiplatelet drugs, minor adverse events (gastrointestinal symptoms and spontaneous purpura) occurred in 6 of 61 patients, but major side effects, including severe gastrointestinal bleeding and intracranial hemorrhage, were not seen. On the other hand, two patients who were taking edoxaban but not antiplatelet drugs experienced fatal bleeding: one intestinal bleeding and one intracranial hemorrhage. Among patients receiving neither antiplatelet drugs nor anticoagulants, two patients died of intracranial hemorrhage and two patients experienced gastrointestinal bleeding requiring hospital admission.

## Discussion

This study demonstrated that antiplatelet therapy started prior to home-care service, especially aspirin therapy, was related to favorable survival among patients with a mean age of 79.4 years who received physician home visits.

One possible reason that antiplatelet therapy was associated with better outcomes is that patients who received physician home visits had a high prevalence of cardiovascular risk factors, resulting in secondary prevention. In other words, a previous physician’s decision to prescribe antiplatelet drugs may have been related to the patient’s underlying cardiovascular risk factors. As we showed sub-analyses, end-stage cancer patients had better outcome from taking antiplatelet agents, whereas non-end-stage cancer patients didn’t. We hypothesized that end-stage cancer patients may have benefits from the agents due to their thrombophilia, which was frequently observed in terminal cancer patients. In contrast, the finding that non-end-stage patients had similar mortality regardless of any antiplatelet agents may come from the lack of power due to limited number of deaths. Another possible reason is that intensive care by home visits from both physicians and nurses, which are usually provided together by our hospital, may have improved the patient’s adherence to medication and the physician’s evaluation of the patient’s condition.

Each patient received at least two visits by a physician and an average of six visits by nurses every month [14]. Under the Japanese healthcare services, the physicians and nurses visited the patients and provided care, including emergency care 24 h daily for 365 days a year. The collaboration of pharmacists may have had additional effects on drug adherence. Thus, a multidisciplinary approach to patient care may be synergistic.

Of the patients in the study, 314 (37.7%) had end-stage cancer. Meta-analyses of previous randomized prevention trials of aspirin showed a protective effect of aspirin on cancer-related death [15] and death from the metastatic spread of cancer [16].

The presence of cancer is now considered an independent risk factor for cardiovascular disease via the production of proinflammatory cytokines and chemokines by cancer cells [17, 18]. Given that patients receive intensive home care in Japan, the benefits of antiplatelet therapy may still exceed the harm from the therapy among patients with cancer.

Apart from the secondary prevention of cardiovascular diseases, many patients were receiving antiplatelet drugs throughout the study period. Although we cannot identify other primary reasons for prescribing antiplatelet drugs by some previous physicians, the patients’ risk factors for cardiovascular diseases might be relevant to the medication.

Patients taking antiplatelet drugs had only a few minor adverse events and no major bleeding events. On the other hand, two patients receiving direct oral anticoagulants had fatal adverse events. Among the patients receiving neither antiplatelet drugs nor anticoagulants, two patients died of intracranial hemorrhage and two patients experienced gastrointestinal bleeding that needed hospital admission.

Antiplatelet drugs may be tolerable in patients receiving home visit care, so that the home visit physician may not need to discontinue these drugs because of concern for unexpected, severe side effects. Therefore, it would be better not to stop the prescription for antiplatelet drugs after the initiation of physician home visits.

Our study has some limitations. First, because it was a retrospective study confined to the period of home visits, detailed prescription information, including the dates of starting and stopping prescriptions, was lacking. Some patients may have stopped taking antiplatelet drugs and others may have started taking antiplatelet drugs during follow-up. Therefore, among patients who were prescribed antiplatelet drugs by a previous physician prior to physician home visits, we cannot compare the outcome between patients who continued antiplatelet therapy and those who stopped antiplatelet therapy. Second, our study was conducted in Japan, where patients can receive intensive home healthcare services, and the results may not be generalizable to patients in other circumstances. Third, the mechanisms by which antiplatelet drugs could reduce mortality of these home-care patients remain unclear. A prospective, multicenter intervention study would be necessary to investigate this question. In addition, limited number of our patients took antiplatelet drugs (61 out of 815 patients). This inequivalent proportion of taking antiplatelet drugs may bias our results. Equally balanced proportion of exposure would be required to evaluate unbiased association. Moreover, although there were no previous reports about prescription rates of any antiplatelet agents for patients requiring physician home visit, our findings which may be biased due to unbalanced proportion of antiplatelet agents might not generalize to other situation.

In conclusion, the continuous prescription of antiplatelet drugs may have beneficial effects on mortality among patients who receive physician home visits.

## Conclusions

The continuous prescription of antiplatelet drugs may have beneficial effects on mortality among patients who receive physician home visits.

## Supplementary information

**Additional file 1:** Hazard ratio (HR) for mortality with antiplatelet therapy status and all adjusted variables from Cox proportional hazard model.

**Additional file 2:** Hazard ratio (HR) for mortality among patients taking antiplatelet agents compared with those not taking antiplatelet agents, excluding patients who lost follow up or died within 2 weeks from baseline.

## Data Availability

The datasets used and/or analysed during the current study are available from the corresponding author on reasonable request.
