# Supplementary material for: Effect of continuation of antiplatelet therapy on survival in patients receiving physician home visits
Source: BMC Geriatr. 2019 Dec 23;19:366. doi: 10.1186/s12877-019-1394-6 (PMC6929486; doi:10.1186/s12877-019-1394-6)
Supplement: Supplementary file 1 — Additional file 1: Hazard ratio (HR) for mortality with antiplatelet therapy status and all adjusted variables from Cox proportional hazard model. [file 12877_2019_1394_MOESM1_ESM.docx]

Supplementary 1. Hazard ratio (HR) for mortality with antiplatelet therapy status and all adjusted variables from Cox proportional hazard model.

|  | Adjusted HR (95% CI) | | |
| --- | --- | --- | --- |
|  | Model 1 | Model 2 | Model 3 |
| Any antiplatelet therapy | **0.28 (0.15–0.53)** | **0.35 (0.18–0.65)** | **0.33 (0.17–0.65)** |
| Age | 1.00 (0.99**–**1.01) | 1.01 (0.99**–**1.01) | 1.01 (0.99**–**1.01) |
| Gender, male | **1.43 (2.27–1.75)** | 1.18 (0.96–1.45) | 1.16 (0.94–1.43) |
| Activities of daily living |  |  |  |
| Ambulation |  |  |  |
| Confined to bed | Reference | Reference | Reference |
| Confined to wheelchair | 0.68 (0.38**–**1.23) | 0.98 (0.54**–**1.76) | 1.04 (0.57**–**1.90) |
| Walks with assistance | 0.97 (0.53**–**1.78) | 1.41 (0.77**–**2.59) | 1.53 (0.83**–**2.85) |
| Walkings independently | 1.26 (0.69**–**2.30) | 1.72 (0.95**–**3.12) | **1.90 (1.04–3.49)** |
| Eating |  |  |  |
| Unable to eat | Reference | Reference | Reference |
| Eats with assistance | 1.04 (0.45**–**2.52) | 0.84 (0.35**–**2.01) | 0.71 (0.28**–**1.76) |
| Eats independently | 1.04 (0.43**–**2.52) | 0.98 (0.39**–**2.44) | 0.84 (0.32**–**2.16) |
| Toileting |  |  |  |
| Needs complete assistance | Reference | Reference | Reference |
| Needs some assistance | **1.80 (1.02–3.19)** | 1.61 (0.91**–**2.86) | **1.88 (1.04–3.43)** |
| Independent | 1.80 (0.99**–**3.20) | 1.62 (0.90**–**2.91) | **2.08 (1.13–3.83)** |
| Primary disease requiring physician home visits | ― |  |  |
| Congenital anomalies |  | 0.44 (0.05**–**3.62) | 0.44 (0.05**–**3.64) |
| Diseases of the circulatory system |  | 0.65 (0.29**–**1.46) | 0.66 (0.28**–**1.53) |
| Diseases of the digestive system |  | 1.48 (0.57**–**3.86) | 1.03 (0.37**–**2.82) |
| Diseases of the genitourinary system |  | 1.01 (0.36**–**2.81) | 0.90 (0.31**–**2.62) |
| Diseases of the musculoskeletal system and connective tissue |  | 0.77 (0.31**–**1.95) | 0.82 (0.32**–**2.09) |
| Diseases of the nervous system and sense organs |  | 0.52 (0.22**–**1.27) | 0.55 (0.23**–**1.35) |
| Diseases of the respiratory system |  | 1.86 (0.82**–**4.23) | 1.95 (0.83**–**4.62) |
| Diseases of the skin and subcutaneous tissue |  | 0.32 (0.04**–**2.66) | ― |
| Endocrine, nutritional, and metabolic diseases |  | 1.10 (0.41**–**2.98) | 1.06 (0.37**–**3.07) |
| Infectious and parasitic diseases |  | 2.23 (0.78**–**6.41) | 1.48 (0.49**–**4.46) |
| Injuries and poisoning |  | 0.57 (0.19**–**1.70) | 0.53 (0.18**–**1.63) |
| Mental illness |  | 0.56 (0.24**–**1.35) | 0.63 (0.26**–**1.53) |
| Neoplasms |  | **3.94 (1.84–8.43)** | **2.46 (1.11–5.46)** |
| Unknown |  | 1.00 (1.00–1.00) | 1.00 (1.00–1.00) |
| Charlson comorbidity index | ― | ― |  |
| Acute myocardial infarction |  |  | 1.36 (0.42**–4.43**) |
| Congestive heart failure |  |  | 0.98 (0.61**–**1.58) |
| Peripheral vascular disease |  |  | 1.47 (0.19**–**11.1) |
| Cerebral infarction |  |  | 1.10 (0.63**–**1.93) |
| Cerebral bleeding |  |  | 0.49 (0.16**–**1.49) |
| Dementia |  |  | 0.62 (0.37**–**1.04) |
| Pulmonary disease |  |  | 1.14 (0.71**–**1.82) |
| Connective tissue disorder |  |  | ― |
| Peptic ulcer |  |  | 0.57 (0.13**–**2.47) |
| Mild to moderate liver disease |  |  | **2.24 (1.06–4.72)** |
| Diabetes |  |  | 1.13 (0.68**–**1.87) |
| Paraplegia |  |  | ― |
| Renal disease |  |  | 1.40 (0.77**–**2.52) |
| Diabetes with complications |  |  | 1.11 (0.14**–**9.04) |
| Cancer |  |  | **1.73 (1.25–2.39)** |
| Leukemia |  |  | 4.90 (0.52**–**46.5) |
| Malignant lymphoma |  |  | 1.85 (0.81**–**4.24) |
| Severe liver disease |  |  | 1.21 (0.51**–**2.92) |
| Metastatic cancer |  |  | **1.53 (1.13–2.09)** |
| HIV |  |  | 1.00 (1.00–1.00) |

Model 1 includes patient’s demographic characteristics and activities of daily living (ADL) for adjustment; model 2 includes primary disease requiring home visiting care in addition to model 1; and model 3 includes the Charlson comorbidity index in addition to model 2.

Patients without each antiplatelet drug included those who took other antiplatelet drugs than the main drug. (e.g. patients without aspirin composed from those who didn’t take any antiplatelet drugs and those who took either clopidogrel or cilostazol.)

Boldface indicates p value < 0.05.
