# Supplementary material for: Effect of continuation of antiplatelet therapy on survival in patients receiving physician home visits
Source: BMC Geriatr. 2019 Dec 23;19:366. doi: 10.1186/s12877-019-1394-6 (PMC6929486; doi:10.1186/s12877-019-1394-6)
Supplement: Supplementary file 2 — Additional file 2: Hazard ratio (HR) for mortality among patients taking antiplatelet agents compared with those not taking antiplatelet agents, excluding patients who lost follow up or died within 2 weeks from baseline. [file 12877_2019_1394_MOESM2_ESM.docx]

Supplementary 2. Hazard ratio (HR) for mortality among patients taking antiplatelet agents compared with those not taking antiplatelet agents, excluding patients who lost follow up or died within two weeks from baseline

| Therapy | Adjusted HR (95% CI) |
| --- | --- |
| Any antiplatelet therapy v.s. Without antiplatelet therapy | **0.39 (0.19 –0.79)** |
| Aspirin v.s. Without aspirin | **0.41 (0.19–0.90)** |
| Clopidogrel v.s. Without clopidogrel | 0.39 (0.09–1.66) |
| Cilostazol v.s. Without cilostazol | 1.34 (0.29–6.29) |

Model includes patient’s demographic characteristics, activities of daily living (ADL), primary disease requiring home visiting care and the Charlson comorbidity index.

Patients without each antiplatelet drug included those who took other antiplatelet drugs than the main drug. (e.g. patients without aspirin composed from those who didn’t take any antiplatelet drugs and those who took either clopidogrel or cilostazol.)

Boldface indicates p value < 0.05.
